# Co-Selection of Antibiotic and Disinfectant Resistance in Bacteria Isolated from Reusable Blood Collection Tourniquets: Implications for Infection Control

**DOI:** 10.3390/jcm15072742

**Published:** 2026-04-04

**Authors:** Julia Szymczyk, Marta Jaskulak, Katarzyna Zorena, Wioletta Mędrzycka-Dąbrowska

**Affiliations:** 1 Department of Anesthesiology and Intensive Care, University Clinical Center, Dębinki 7, 80-952 Gdansk, Poland; szymczykjulia@gumed.edu.pl; 2 Department of Immunobiology and Environmental Microbiology, Faculty of Health Sciences, Medical University of Gdansk, 80-211 Gdansk, Poland; marta.jaskulak@gumed.edu.pl (M.J.); katarzyna.zorena@gumed.edu.pl (K.Z.); 3 Department of Anesthesiology Nursing & Intensive Care, Faculty of Health Sciences, Medical University of Gdansk, 80-211 Gdansk, Poland

**Keywords:** antibiotic, disinfectant resistance, infection control, blood collection tourniquets

## Abstract

**Background:** Reusable tourniquets are widely used across clinical settings, yet their role as reservoirs of microbial contamination and antimicrobial resistance remains poorly characterized. **Methods:** In this cross-sectional study, 53 polyester–elastane tourniquets were collected from an Emergency Department (SR) and Operating Theater (SBO) over a 28-day period to assess bacterial burden and resistome composition. A 180-target qPCR panel targeting antibiotic and disinfectant resistance determinants was used. qPCR analysis identified 112 distinct resistance genes across all samples, with SR tourniquets harboring significantly richer resistomes than SBO (median 34 vs. 15 genes; *p* < 0.001). Efflux pump- and disinfectant-associated genes were pervasive, and β-lactamase and quinolone-resistance determinants increased over time in SR samples. **Results:** Principal component analysis showed clear segregation of resistome profiles by clinical unit and progressive enrichment over time. These findings indicate that reusable, porous tourniquets can accumulate extensive resistance gene profiles under routine clinical use, particularly in high-contact environments. **Conclusions:** Enhanced decontamination strategies, development of new materials or transition to single-use alternatives may be necessary to mitigate their potential contribution to environmental antimicrobial resistance in hospitals.

## 1. Introduction

Healthcare-associated infections (HAIs) represent one of the most serious challenges in modern medicine. According to the latest WHO 2024 report, on average, 7 out of 100 patients in high-income countries and 15 out of 100 patients in low- and middle-income countries acquire at least one HAI during their hospital stay [1]. Across Europe, this issue affects 4.3 million patients annually in EU/EEA hospitals, with respiratory infections accounting for 29.3% of cases [2,3]. Alarmingly, 23.6% of all sepsis cases treated in hospitals are healthcare-associated, rising to 48.7% for organ-dysfunction sepsis in adult intensive care units [1].

Antimicrobial resistance (AMR) significantly amplifies this burden, currently incurring an additional global healthcare cost of USD 66 billion per year, projected to reach USD 159 billion by 2050 without intervention [4,5,6,7]. Particularly problematic are the so-called ESKAPE pathogens—*Enterococcus faecium*, *Staphylococcus aureus*, *Klebsiella pneumoniae*, *Acinetobacter baumannii*, *Pseudomonas aeruginosa*, and *Enterobacter* species—which are responsible for a large proportion of healthcare-associated infections and are known for their ability to rapidly acquire multidrug resistance. At the patient level, resistant infections are associated with 29.3% higher hospitalization costs and 23.8% longer length of stay compared to susceptible infections. In developing countries, the average extra AMR-related cost per hospital is approximately USD 650,000 annually [8].

Reusable medical devices—such as blood pressure cuffs and pulse oximeters—act as important fomites, facilitating the transmission of multidrug-resistant pathogens. Studies report that 71% of blood pressure cuffs in Emergency Departments are contaminated prior to disinfection, while 91% of pulse oximeters in intensive care units remain contaminated, 68% of which harbor pathogens like *Acinetobacter baumannii*, *Klebsiella pneumoniae*, and *Pseudomonas aeruginosa*. A 2024 study from Gdańsk documented a mean bioburden of 545 CFU/cm^2^ on tourniquets in the admission ward and 101 CFU/cm^2^ in the Operating Theater, representing the first Polish data on this issue [9].

A critical factor in AMR development and dissemination is the co-selection of antibiotic and disinfectant resistance. Research shows that 94% of antibiotic-resistant bacteria also resist at least one disinfectant. Class 1 integrons play a central role by carrying both antibiotic-resistance genes and *qacE*/*qacEΔ1* genes conferring resistance to quaternary ammonium compounds [10]. These mobile elements facilitate horizontal gene transfer, spreading multidrug resistance in hospital environments.

Biofilm formation on device surfaces further complicates infection control. Biofilms shield bacteria from desiccation and antimicrobials while promoting gene exchange [11,12]. As an example, endoscopic equipment has been implicated in outbreaks of carbapenemase-producing Enterobacteriaceae due to disinfection failures that allow biofilm development.

In clinical and environmental diagnostics, quantitative PCR (qPCR) is widely used for rapid detection of antibiotic-resistance genes and surveillance of antimicrobial resistance in healthcare environments [13].

Given the global burden of HAIs and the increasing dissemination of antimicrobial resistance, identifying environmental reservoirs of resistance within healthcare settings is essential for effective infection prevention and control strategies. This study, framed within the One Health approach—recognizing the interconnected health of humans, animals, and ecosystems—aims to fill the gap in comprehensive molecular characterization of co-selection mechanisms on reusable blood collection tourniquets [14].

## 2. Materials and Methods

### 2.1. Study Design and Setting

This study was designed as an observational cross-sectional environmental study evaluating reusable blood collection tourniquets collected from hospital clinical units. Samples were collected over a defined study period to characterize the presence of antibiotic resistance genes associated with these devices in the hospital environment. Because the study focused on environmental samples rather than patient outcomes, no follow-up period was applicable. The study was conducted between June and July 2025 in Gdansk, Poland. The study protocol followed the Strengthening the Reporting of Molecular Epidemiology for Infectious Diseases (STROME-ID) guidelines, an extension of the STROBE statement [15]. The study was registered at ClinicalTrials.gov (NCT06566495).

### 2.2. Sampling Strategy

Sample collection was carried out at a single tertiary care facility located in Northern Poland. Reusable tourniquets made of polyester–elastane fabric were obtained from two clinical units: the Emergency Department (labeled “SR”) and the Operating Theater (labeled “SBO”). Inclusion criteria comprised reusable tourniquets actively used in these departments. Exclusion criteria included single-use tourniquets, tourniquets made of latex, thermoplastic elastomer (TPE), polyurethane (PU), synthetic rubber, or tourniquets from other hospital units. Sampling was performed at three timepoints: baseline (indefinite), after 14 days, and after 28 days of use. The sampling design is summarized in Table 1.

### 2.3. Sample Collection and Processing

Each tourniquet was collected aseptically and transported to the laboratory at the Department of Immunobiology an Environmental Microbiology, Faculty of Health Sciences, Medical University of Gdansk, Poland. Plastic elements were removed, and the fabric part was placed in a sterile bag with nutrient broth and homogenized using a BagMixer 400 (Interscience, Nom la Bretèche, France). The resulting suspension was inoculated onto microbiological media (Columbia agar, MacConkey agar, KinB) and incubated at 37 °C under aerobic conditions for 24 h. Colony-forming units (CFU/cm^2^) were counted. Cultures in nutrient broth were transferred to sterile 15 mL Falcon tubes (Genoplast, Pomorskie, Poland) and centrifuged at 5000× *g* for 10 min, and pellets were stored at −80 °C.

### 2.4. Microbiological Analysis—Culture-Based Study and 16S rRNA Sequencing

After incubation, the number of colony forming units (CFU) was determined by counting visible colonies on the agar plates. In a separate analysis, DNA was extracted from the samples using ExtractMe Total DNA purification columns (Blirt, Gdańsk, Poland) according to the manufacturer’s protocol. DNA concentration and purity were measured with a NanoDrop spectrophotometer (Thermo Scientific, Waltham, MA, USA). The resulting genetic material was preserved in nutrient broth at −80 °C. The V3–V4 region of the 16S rRNA gene was amplified and sequenced using next-generation sequencing (NGS) technology (Makrogen, Seoul, Republic of Korea). Obtained sequence data were filtered and taxonomically assigned using BLAST against the NCBI 16S rRNA database. The results of 16S rRNA Sequencing were published in a separate study [16].

### 2.5. Detection of Antibiotic and Disinfectant Resistance Genes by qPCR

DNA extracted from 53 reusable tourniquet samples was analyzed for the presence of antibiotic and disinfectant resistance genes using quantitative real-time PCR (qPCR). Primer sequences were designed in Primer3Pro based on consensus regions identified from NCBI BLAST reference sequences. Each primer pair targeted a unique, conserved region within the corresponding resistance gene to avoid off-target amplification. Primers were 18–24 bp in length, with melting temperatures (Tm) of 58–62 °C, GC content of 40–60%, and predicted amplicon sizes of 80–150 bp. In silico validation included BLAST specificity checks against the NCBI nt database, exclusion of primer–dimer and hairpin structures, and alignment against closely related ARG variants to ensure discriminatory power. All primers were synthesized by a commercial vendor (Genomed, Warsaw, Poland) and reconstituted to 100 µM stock solutions before preparation of 10 µM working solutions. Primer performance was verified empirically using gene-positive control templates, and amplification efficiencies were calculated from standard curves generated using 10-fold serial dilutions (efficiency acceptance range: 90–110%, R^2^ > 0.98).

qPCR reactions were performed in 10 µL volumes containing 5 µL of MP qPCR Master Mix (2×) (EURx, Gdańsk, Poland), 0.4 µM of each primer, 2 µL of template DNA (normalized to 2–5 ng per reaction), and nuclease-free water. All assays were run in singleplex format to avoid primer interaction and ensure consistent melt-curve interpretation. Reactions were performed in a Mic qPCR Cycler (Bio Molecular Systems, Gold Coast, Australia) with the following cycling conditions: initial denaturation at 95 °C for 2 min, followed by 40 cycles of 95 °C for 5 s and 60 °C for 20 s. Fluorescence was acquired at the end of each extension step. Amplification specificity was confirmed by high-resolution melt-curve analysis (70–95 °C, 0.1 °C/s ramp), and only reactions displaying single melt peaks within ±0.5 °C of the expected Tm were considered specific. Ct < 35 was used as the threshold for a positive detection; reactions with Ct 35–38 or atypical melt profiles were repeated, and only confirmed positives were retained. All samples and controls were run in technical duplicates; discordant replicates were re-assayed. The full list of chosen ARGs is presented in Table 2. No PCR amplicons were sequenced; all analyses were based on qPCR presence/absence detection. Detection of resistance genes using qPCR indicates the presence of genetic material but does not necessarily confirm the presence of viable bacteria carrying these genes. The detected DNA may originate from non-viable cells, extracellular DNA, or environmental contamination. Therefore, the results should be interpreted as evidence of the presence of resistance gene signatures rather than direct proof of viable resistant microorganisms on the analyzed surfaces. The resistance gene panel was designed to cover a broad spectrum of clinically relevant antibiotic resistance genes commonly reported in healthcare environments, including genes associated with β-lactam, macrolide, aminoglycoside, tetracycline, and sulfonamide resistance. In addition, genes related to disinfectant resistance and multidrug efflux systems were included to explore potential co-occurrence patterns between antibiotic and disinfectant resistance determinants in hospital settings. The primer panel was adapted from previously published assays targeting clinically relevant antibiotic resistance genes. Additional primers were designed using Primer3 software (version 4.1.0) to expand the panel and include resistance determinants associated with disinfectant resistance and multidrug efflux systems. Prior to analysis, primer performance was evaluated to ensure specificity and reliable amplification. Primer pairs showing poor amplification efficiency or non-specific amplification signals were excluded from further analysis. Only primer sets that met the predefined quality criteria were retained in the final resistance gene panel used in this study. Negative extraction controls were included during DNA extraction to monitor potential contamination introduced during sample processing. To minimize the risk of contamination during qPCR analysis, reactions were prepared in a dedicated clean area using sterile consumables and standard laboratory contamination-control procedures. Because the study focused on the presence or absence of resistance genes rather than quantitative abundance, the data were not normalized to total bacterial load (e.g., 16S rRNA gene copy number). The 180-gene panel was assembled using a combination of previously published resistance targets reported in environmental and clinical resistome studies and additional targets selected specifically for this study to capture disinfectant resistance and multidrug efflux determinants relevant to healthcare settings.

### 2.6. Statistical Analysis

The obtained data was analyzed using R programming language (version 3.6.0) and RStudio. Class-level summaries (e.g., β-lactamases, carbapenemases, efflux, disinfectant-associated genes) were derived by aggregating genes according to functional annotation, and median richness per class was compared between SR and SBO. Heatmaps of gene detection patterns were generated in BLAST using matplotlib. To assess patterns of co-occurrence among resistance determinants, pairwise correlation coefficients were computed from binary presence/absence data, producing both a complete 180 × 180 correlation matrix and a focused 30-gene matrix based on the most prevalent targets.

Principal component analysis (PCA) was performed on the full 180-gene presence/absence matrix to characterize global variation in resistome profiles. PCA was implemented in Python using scikit-learn, and the first two principal components were examined to evaluate separation between clinical units and temporal shifts in resistome composition. Loadings were inspected to identify the ARGs contributing most to observed variance. Supplementary metrics included pairwise gene-correlation distributions, summary statistics (mean, median, minimum, maximum correlation), and co-occurrence network interpretation. Groupwise comparisons between clinical units and timepoints were performed using non-parametric tests (Mann–Whitney U test for two groups and Kruskal–Wallis test for multiple groups), because resistome richness values did not assume normal distribution. *p*-values < 0.05 were considered statistically significant. For statistical comparisons, the analyses were performed on aggregated variables such as resistome richness (number of detected resistance genes per sample) rather than on individual binary gene presence/absence values. These variables were treated as continuous measures representing the overall resistome diversity within samples.

Non-metric multidimensional scaling (NMDS) was performed to explore patterns in the distribution of resistance genes across samples based on the binary presence/absence matrix. The analysis used the Jaccard distance metric, which is appropriate for binary ecological data. The resulting ordination revealed clustering patterns consistent with the PCA results. The stress value of the NMDS ordination was 0.11, indicating an acceptable representation of the data in two-dimensional space.

For analyses involving multiple gene targets, *p*-values were adjusted for multiple comparisons using the Benjamini–Hochberg false discovery rate (FDR) correction to control for type I error. Adjusted *p*-values (FDR) below 0.05 were considered statistically significant.

### 2.7. Ethical Considerations

The study was approved by the Independent Bioethics Committee for Scientific Research at the Medical University of Gdańsk (decision number KB/45/2024 of 29 February 2024).

## 3. Results

### 3.1. Detection of Antibiotic and Disinfectant Resistance Genes on Tourniquets

A total of 180 primer pairs targeting antibiotic and disinfectant resistance determinants were tested across 53 reusable tourniquet samples. The quantitative PCR resistome analysis identified contamination with antibiotic and disinfectant resistance determinants across the tourniquets. Of the 180 primer pairs, 112 distinct genes (62.9%) were detected at least once overall. The resistome composition varied strongly between clinical units. SR tourniquets carried a median of 34 ARGs per sample, compared with 15 in SBO samples (*p* < 0.001).

**Figure 1:**
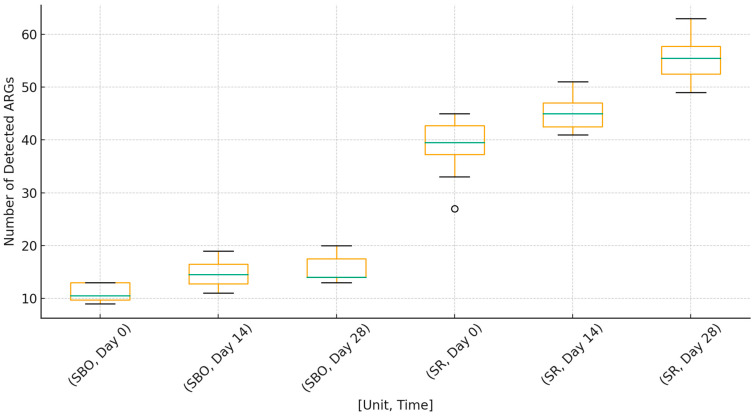
ARG dissemination per sample by unit and timepoints (SBO—Operating Theater tourniquets, SR—Emergency Department tourniquets). Results shown as average with median, quartile and standard deviation.

Moreover, SR samples demonstrated progressive resistome enrichment over time, with median gene counts increasing from 22 at baseline to 31 after 14 days and 39 after 28 days (trend test, *p* < 0.01). No corresponding accumulation was observed in SBO samples (*p* = 0.41). SR tourniquets carried significantly more genes than SBO (median 34 vs. 15, *p* < 0.001). The overall ARGs loads across different units and timepoints are presented in Figure 1.

### 3.2. Resistome Composition by ARG Class

Across all samples, efflux-associated determinants constituted the most abundant and diverse class, with 26 of 28 targeted genes detected in the cohort. Disinfectant resistance genes, including *qac* and *smr* families, were similarly widespread, detected in 18 of the 19 primer targets and found at high prevalence in SR tourniquets. In contrast, glycopeptide and carbapenemase genes were identified infrequently; *vanA* and *vanB* were detected in only a minority of SR samples, while *blaKPC-*, *blaNDM-*, and *blaOXA-48*-like genes were restricted to sporadic detections. β-lactamase and tetracycline resistance determinants were broadly distributed across samples, with 21/29 β-lactamase genes and 19/22 tetracycline genes amplified at least once. Taken together, overall, the efflux and disinfectant classes showed the highest richness and prevalence. Carbapenemases (class 2) were rare and almost exclusively found in SR samples. β-lactamases and tetracycline genes were widespread. In summary, SR tourniquets host a diverse resistome dominated by efflux- and disinfectant-associated genes, consistent with heavy handling and disinfectant pressure (Table 3).

### 3.3. Most Prevalent ARGs from a Gene-Level View and Principal Component Analysis

On a singular-gene level, the most commonly detected were the *qacC* gene (disinfectant resistance) and *mdf*/*emr* (multidrug efflux) genes. Carbapenemases (*oxa48*, *blaKPC*) were rare but present. The most commonly detected genes on all 53 tourniquets with ARGs are presented in Table 4.

By unit, SR tourniquets consistently carried more ARGs, especially disinfectant efflux genes (*qacC*). The most prevalent ARGs across all samples are presented in Table 4 and by unit comparison in Table 5. SR samples consistently formed a high-prevalence block with dense ARG co-occurrence, whereas SBO samples exhibited sparse and discontinuous amplification patterns. The differences in the prevalence of the three most detected ARGs between SR and SBO tourniquets were significant (*p* < 0.05).

**Figure 2:**
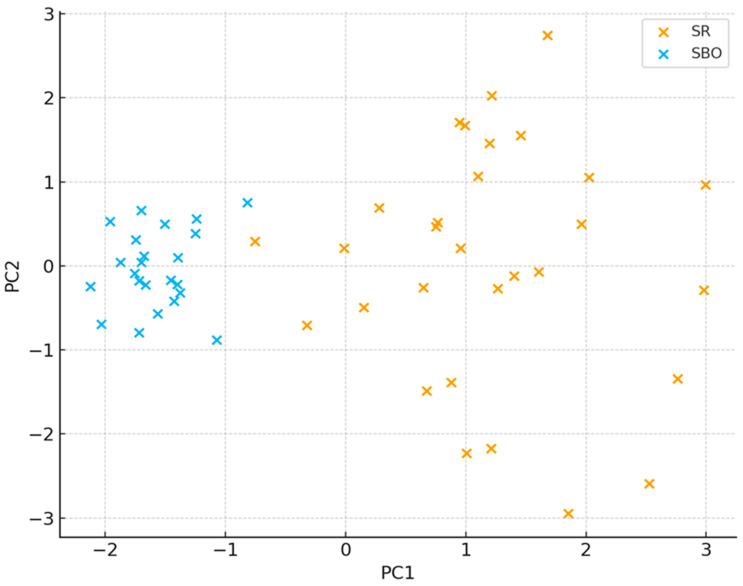
Resistome Ordination (PCA/NMDS). PC1 explains 33% of the variance, and PC2 explains 14%. Clear separation between SR and SBO samples is observed along PC1, while time progression (0 → 14 → 28 days) is visible along PC2. NMDS stress value = 0.11.

Principal component analysis of the 180-gene presence/absence matrix demonstrated clear segregation of resistome profiles between the two clinical units (Figure 2). PC1, accounting for 33% of total variance, separated SR from SBO, while PC2 (14% of variance) represented the temporal component of resistome expansion, particularly in SR samples. NMDS ordination supported the separation between units, confirming the clustering observed in the PCA.

### 3.4. Co-Occurrence Networks and Gene Modules

**Figure 3:**
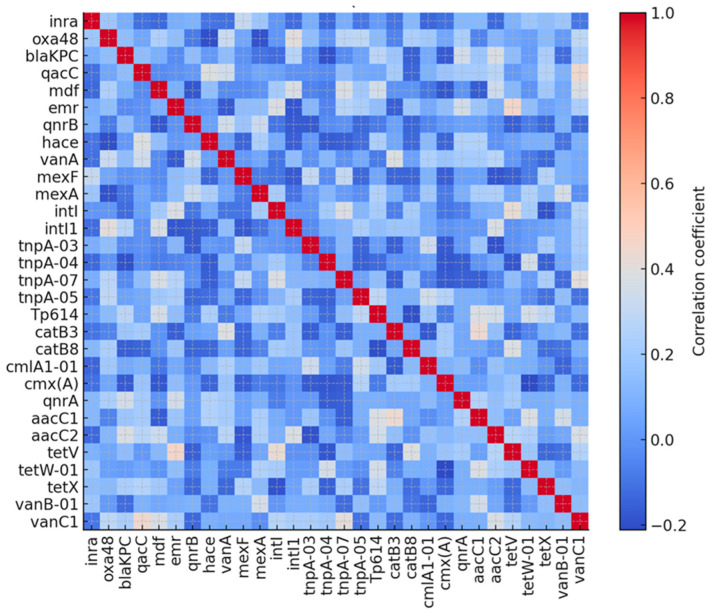
Co-occurrence correlation matrix for top 30 genes.

Pairwise co-occurrence of the 180 resistance targets, assessed using a correlation matrix of the presence/absence profiles, revealed a structured resistome rather than a random assemblage of genes. In the full 180 × 180 matrix, most gene pairs showed weak but positive correlation, with a mean correlation coefficient of approximately 0.07, while values ranged from about −0.28 to 0.50 in the subset of the 30 most prevalent genes. Inspection of the top 30 genes heatmap demonstrated blocks of strongly co-occurring determinants, particularly among efflux pump genes and disinfectant-resistance genes and between several β-lactamase and quinolone-resistance targets (Figure 3). These patterns suggest non-random co-occurrence of resistance determinants, potentially reflecting shared ecological pressures or recurrent association within bacterial communities exposed to repeated disinfectant stress. Overall, the correlation analysis supports the presence of distinct ARG constellations on reusable tourniquets, rather than isolated single determinants, supporting the view that these devices may act as reservoirs of recurrently co-occurring resistance gene clusters.

To further illustrate the structure of gene co-occurrence, we constructed a correlation-based network using genes with |r| ≥ 0.4. The resulting graph revealed three prominent clusters. The first comprised primarily disinfectant and multidrug efflux determinants, including *qacC*, *qacEΔ1*, *mdf*, *emrE*, *mexA*, *mexF*, *acrA-01*, *ceoA* and *cmr*. A second cluster grouped β-lactamase and quinolone resistance genes such as *blaKPC*, *blaOXA48*, *NDM*, *blaTEM*, *qnrB* and *qnrA*. A third cluster contained mobile-element-associated genes (*intI*, *intI1*, *tnpA-03*, *tnpA-04*, *Tp614*), which frequently connected the other two modules. This pattern may indicate the potential co-occurrence of disinfectant and antibiotic resistance determinants; however, co-localization on shared genetic platforms cannot be confirmed without genomic sequencing. Overall, in the correlation analysis, three major modules have emerged: (1) efflux-disinfectant module with *qacC*, *qacE*, *smr*, *mdf(A)*, *emrB*, *mexA/F* exhibiting high internal correlation (r = 0.62–0.88); (2) β-lactam–Quinolone Module with *blaTEM*, *blaOXA-like*, *blaCTX-M-like*, *qnrB*, and *qnrS* exhibiting moderate correlation (*r* = 0.44–0.71); (3) mobile genetic elements module with *intl1*, *intl2*, *tnpA-like* genes exhibiting moderate-to-high internal correlation (0.57–0.79).

Overall, the combined culture, sequencing, and qPCR results indicate that reusable polyester–elastane tourniquets harbor complex, diverse, and dynamically evolving microbial and genetic communities, with particularly high burdens in the Emergency Department. The enrichment of disinfectant and efflux-associated resistance determinants suggests strong selective pressures associated with repeated handling and disinfection practices, while the progressive accumulation of antibiotic resistance genes over time underscores the potential role of reusable fabric medical tools as persistent reservoirs of clinically relevant ARGs.

## 4. Discussion

Healthcare-associated infections (HAIs) remain a major global challenge, and increasing evidence shows that environmental reservoirs within hospitals contribute to the persistence and transmission of antimicrobial-resistant organisms. In this study, we aimed to determine whether reusable blood collection tourniquets serve as overlooked vectors of bacterial contamination and antibiotic resistance genes. Overall, the study provides a characterization of the resistome carried by reusable blood collection tourniquets in a tertiary hospital setting.

The findings highlight the potential of widely used fabric medical accessories to serve as overlooked vectors in the persistence and dissemination of antimicrobial resistance (AMR) within healthcare environments.

The elevated ARG burden observed on SR tourniquets is consistent with their operational context. Emergency Departments represent high-throughput, high-contact clinical environments characterized by rapid patient turnover, intense device handling, and frequent but often suboptimal disinfection cycles due to workflow constraints. In contrast, tourniquets from the Operating Theater are handled within stricter aseptic protocols.

The resistome analysis suggests differential contamination patterns between the analyzed tourniquet types. Across all samples, 112 distinct ARGs were detected, with SR tourniquets carrying more than twice as many ARGs per sample as their SBO counterparts. The magnitude of this difference is notable and reinforces the idea that tourniquets can potentially capture and retain DNA from diverse bacterial communities rather than reflecting a single organism’s genome. The predominance of efflux pumps and disinfectant resistance determinants is especially important. Genes such as *qacC*, *mdf*, *emr*, *mexA*, and *mexF*, which were abundant in our dataset, are commonly associated with repeated exposure to quaternary ammonium compounds (QACs) and alcohol-based disinfectants [17].

Their pervasive presence across SR samples may reflect selective pressures associated with repeated exposure to disinfectants, although the present study does not directly assess the viability of microorganisms or the genetic context of these resistance determinants [15,17]. Similar enrichment of *qac* and small multidrug resistance (*smr*) genes has been reported in hospital isolates and on disinfectant-exposed surfaces, where carriage of these determinants correlates with reduced susceptibility to QAC-based products [18].

When placed in the broader context of hospital environmental resistome studies, our findings are consistent with reports that high-contact surfaces harbor complex communities of ARGs. Metagenomic and qPCR-based surveys of hospital surfaces and air dust have shown widespread detection of resistance determinants, including β-lactamase, carbapenemase, vancomycin, and efflux genes, often in most sampled sites [19]. However, most of these studies focus on fixed surfaces (e.g., bed rails, worktops, sinks, handwash stations) or air dust and do not specifically address small, handheld reusable items. This project fills this gap by demonstrating that a single category of frequently reused, fabric-based equipment can concentrate a resistome that is comparable in diversity to that described in larger environmental reservoirs. In particular, the high prevalence of disinfectant- and efflux-associated genes on tourniquets echoes observations from hospital surface and disinfectant contamination studies, where QAC-tolerant *Serratia*, *Pseudomonas*, and *Staphylococcus* isolates have been linked to persistent environmental colonization despite routine cleaning [20,21].

The identification of β-lactamase and quinolone-resistance determinants in both units and their temporal increase in SR raise important concerns. While carbapenemase genes were rare, their detection—albeit sporadically—on reusable tourniquets cannot be dismissed. Even at low prevalence, such genes pose disproportionate risk given their association with multidrug-resistant pathogens [11]. The co-occurrence modules identified in the correlation network analysis indicate that ARGs on tourniquets are not randomly distributed but tend to appear in consistent association patterns. However, because the present study did not include genomic sequencing or plasmid analysis, it is not possible to determine whether these genes are physically linked on the same mobile genetic elements. The grouping of qnr, blaTEM, and integrase-associated genes may indicate potential co-occurrence patterns consistent with plasmid- or integron-mediated dissemination described in other studies, although direct genetic linkage cannot be confirmed without genomic sequencing [22].

From an infection control standpoint, our findings align with emerging evidence that the choice of tourniquet material and reprocessing strategy influences contamination. For example, silicone tourniquets have been associated with lower bacterial loads and easier disinfection compared with conventional fabric devices in clinical use [23]. Our data imply that, beyond simply reducing CFU counts, such material changes could also diminish the environmental resistome, particularly for disinfectant- and efflux-related genes that appear to be strongly selected on porous fabric surfaces under current cleaning practices. Together with recent reviews arguing that reusable tourniquets represent underappreciated vectors of AMR transmission, our results support reconsideration of device design, usage policies, and reprocessing protocols [9,24].

Overall, tourniquets, though often considered low-risk medical devices, are used across nearly every patient encounter and frequently travel between clinical spaces. Their widespread handling, fabric construction, and inconsistent disinfection create conditions that support both microbial growth and ARG accumulation. While our study does not directly address transmission events, the presence of clinically relevant resistance determinants on devices that contact the skin of multiple patients raises the possibility of indirect transfer of resistance determinants. Environmental reservoirs enriched with antimicrobial resistance genes may contribute to the background resistome of healthcare settings, increasing the probability of horizontal gene transfer to opportunistic pathogens. The dynamics observed—particularly the rapid enrichment of ARGs within 28 days—suggest that routine cleaning is insufficient to fully disrupt microbial and genetic persistence and that reusable tourniquets may serve as long-term environmental reservoirs contributing to background AMR pressure within hospitals.

### 4.1. Implications for Infection Control

Reusable fabric tourniquets represent underrecognized environmental reservoirs of co-selected antibiotic and disinfectant resistance genes. Routine disinfection alone appears insufficient to prevent progressive resistome enrichment, particularly in high-contact clinical areas. These findings highlight the need to re-evaluate tourniquet materials, reprocessing strategies, and usage policies as part of hospital infection control programs.

### 4.2. Limitations

While a 180-target qPCR panel provides broad coverage across major antibiotic and biocide resistance classes, it cannot capture the full diversity of the resistome detectable by unbiased metagenomic sequencing. Additionally, while qPCR detects gene presence, it does not discriminate between DNA from viable cells, dead cells, or extracellular DNA; thus, quantitative gene abundance should be interpreted in the context of total bacterial load and DNA persistence on porous fabrics.

Despite these limitations, our findings align with growing evidence that environmental surfaces and reusable devices in healthcare settings harbor unexpectedly rich resistomes, shaped by patterns of human contact and disinfection. The strong enrichment of disinfectant resistance determinants underscores the need to re-evaluate cleaning protocols and consider material design in reusable devices. Transitioning to single-use tourniquets, implementing validated sterilization workflows, or adopting surface materials less permissive to microbial adherence may represent viable mitigation strategies. Further metagenomic studies and assessments of intervention efficacy are warranted to clarify the extent to which reusable tourniquets contribute to AMR circulation within hospitals. Because ARG detection was not normalized to total bacterial load, changes over time may reflect both shifts in microbial community composition and accumulation of environmental DNA.

## 5. Conclusions

This study demonstrates that reusable fabric tourniquets function as persistent reservoirs of both bacterial contamination and a diverse array of antibiotic and disinfectant resistance genes. Tourniquets collected from the Emergency Department displayed a substantially richer resistome than those obtained from the Operating Theater, reflecting differences in handling intensity, workflow, and disinfection practices. The detection of over one hundred distinct resistance determinants, including widespread efflux- and quaternary-ammonium-associated genes and sporadic clinically significant β-lactamase and carbapenemase genes, indicates that these devices accumulate complex microbial and genetic signatures over time.

The progressive enrichment of resistance genes during a 28-day period suggests that current disinfection routines are insufficient to prevent the build-up of a resilient resistome on porous, repeatedly handled materials. While the clinical implications of these findings require further investigation, the presence of multidrug resistance determinants on devices used directly on patients highlights the potential for indirect AMR dissemination within healthcare environments.

Collectively, our results support the need to re-evaluate the use of reusable tourniquets in hospital settings. Adoption of single-use alternatives, reusable ones made with new material technologies, or the development of standardized and validated decontamination protocols, may be necessary to reduce the contribution of these devices to microbial persistence and the environmental resistome within clinical units.

## Figures and Tables

**Table 1:** Sampling Design and Observation Timeline in the Emergency Department and the Operating Theater.

No. Sample	Tourniquet Name	Department/Group	Location of Tourniquet Use
1	SR1/1	Emergency Department	Pediatric Room
2	SR2/1	Emergency Department	Pediatric Observation
3	SR3/1	Emergency Department	Adult Observation
4	SR4/1	Emergency Department	Adult Observation
5	SR5/1	Emergency Department	Consultation Area
6	SR6/1	Emergency Department	Resuscitation Area
7	SBO1/1	Operating Theater	Neurologic
8	SBO2/1	Operating Theater	Neurologic
9	SBO3/1	Operating Theater	Orthopedic
10	SBO4/1	Operating Theater	Orthopedic
11	SBO5A/1	Operating Theater	Orthopedic
12	SBO5B/1	Operating Theater	Orthopedic
13	SBO6/1	Operating Theater	Gynecology
14	SBO7/1	Operating Theater	Pediatric
15	SBO8/1	Operating Theater	Pediatric
16	SBO9/1	Operating Theater	General Surgery
17	SBO10/1	Operating Theater	General Surgery
18	SR1/2	Emergency Department	Pediatric Room
19	SR2/2	Emergency Department	Pediatric Observation
20	SR3/2	Emergency Department	Adult Observation
21	SR4/2	Emergency Department	Adult Observation
22	SR5/2	Emergency Department	Consultation Area
23	SR6/2	Emergency Department	Resuscitation Area
24	SBO1/2	Operating Theater	Neurologic
25	SBO2/2	Operating Theater	Neurologic
26	SBO3/2	Operating Theater	Orthopedic
27	SBO4/2	Operating Theater	Orthopedic
28	SBO5A/2	Operating Theater	Orthopedic
29	SBO5B/2	Operating Theater	Orthopedic
30	SBO6A/2	Operating Theater	Gynecology
31	SBO6B/2	Operating Theater	Gynecology
32	SBO7/2	Operating Theater	Pediatric
33	SBO8/2	Operating Theater	Pediatric
34	SBO9A/2	Operating Theater	General Surgery
35	SBO9B/2	Operating Theater	General Surgery
36	SBO10A/2	Operating Theater	General Surgery
37	SBO10B/2	Operating Theater	General Surgery
38	SR1/3	Emergency Department	Pediatric Room
39	SR2/3	Emergency Department	Pediatric Observation
40	SR3/3	Emergency Department	Adult Observation
41	SR4/3	Emergency Department	Adult Observation
42	SR5/3	Emergency Department	Consultation Area
43	SR6/3	Emergency Department	Resuscitation Area
44	SBO1/3	Operating Theater	Neurologic
45	SBO2/3	Operating Theater	Neurologic
46	SBO3/3	Operating Theater	Orthopedic
47	SBO4/3	Operating Theater	Orthopedic
48	SBO5/3	Operating Theater	Orthopedic
49	SBO6/3	Operating Theater	Gynecology
50	SBO7/3	Operating Theater	Pediatric
51	SBO8/3	Operating Theater	Pediatric
52	SBO9/3	Operating Theater	General Surgery
53	SBO10/3	Operating Theater	General Surgery

**Table 2:** Chosen antibiotic and disinfectant resistance genes—primer sequences.

Gene Name	Forward Primer	Reverse Primer	Gene Classification	Antibiotics Resistant/Disinfectant Resistant	Resistance Mechanism
***intI***	GGCATCCAAGCAGCAAG	AAGCAGACTTGACCTGA	Integrase (site-specific recombinase)	None directly (enables capture of resistance cassettes)	Gene-cassette integration/mobilization
***intI1***	CGAACGAGTGGCGGAGGGTG	TACCCGAGAGCTTGGCACCCA	Integrase (site-specific recombinase)	None directly (enables capture of resistance cassettes)	Gene-cassette integration/mobilization
***tnpA-03***	AATTGATGCGGACGGCTTAA	TCACCAAACTGTTTATGGAGTCGTT	Transposase	None directly (can mobilize resistance genes)	DNA transposition/element mobility
***tnpA-04***	CCGATCACGGAAAGCTCAAG	GGCTCGCATGACTTCGAATC	Transposase	None directly (can mobilize resistance genes)	DNA transposition/element mobility
***tnpA-07***	GAAACCGATGCTACAATATCCAATTT	CAGCACCGTTTGCAGTGTAAG	Transposase	None directly (can mobilize resistance genes)	DNA transposition/element mobility
***tnpA-05***	GCCGCACTGTCGATTTTTATC	GCGGGATCTGCCACTTCTT	Transposase	None directly (can mobilize resistance genes)	DNA transposition/element mobility
***Tp614***	GGAAATCAACGGCATCCAGTT	CATCCATGCGCTTTTGTCTCT	Conjugative transposon (mobile element)	Varies; carries resistance cassettes	Horizontal transfer/gene mobilization
***catB3***	GCACTCGATGCCTTCCAAAA	AGAGCCGATCCAAACGTCAT	CAT chloramphenicol acetyltransferase	Chloramphenicol	Drug acetylation = deactivation
***catB8***	CACTCGACGCCTTCCAAAG	CCGAGCCTATCCAGACATCATT	CAT chloramphenicol acetyltransferase	Chloramphenicol	Drug acetylation = deactivation
***cmlA1-01***	TAGGAAGCATCGGAACGTTGAT	CAGACCGAGCACGACTGTTG	Efflux pump (chloramphenicol/florfenicol)	Phenicols	Efflux
***cmx(A)***	GCGATCGCCATCCTCTGT	TCGACACGGAGCCTTGGT	Efflux pump (MFS-type)	Phenicols	Efflux
***qnrA***	AGGATTTCTCACGCCAGGATT	CCGCTTTCAATGAAACTGCAA	Qnr (PMQR protein)	Fluoroquinolones	Target protection (gyrase/topo IV shielding)
***aacC1***	GGTCGTGAGTTCGGAGACGTA	GCAAGTTCCCGAGGTAATCG	AAC (aminoglycoside acetyltransferase)	Aminoglycosides	Drug acetylation = deactivation
***aacC2***	ACGGCATTCTCGATTGCTTT	CCGAGCTTCACGTAAGCATTT	AAC (aminoglycoside acetyltransferase)	Aminoglycosides	Drug acetylation = deactivation
***aacC4***	CGGCGTGGGACACGAT	AGGGAACCTTTGCCATCAACT	AAC (aminoglycoside acetyltransferase)	Aminoglycosides	Drug acetylation = deactivation
***aac(6′)I1***	GACCGGATTAAGGCCGATG	CTTGCCTTGATATTCAGTTTTTATAACCA	AAC (aminoglycoside acetyltransferase)	Aminoglycosides	Drug acetylation = deactivation
***aac(6′)-Ib-01***	GTTTGAGAGGCAAGGTACCGTAA	GAATGCCTGGCGTGTTTGA	AAC (aminoglycoside acetyltransferase)	Aminoglycosides	Drug acetylation = deactivation
***aadA1***	AGCTAAGCGCGAACTGCAAT	TGGCTCGAAGATACCTGCAA	ANT (adenylyltransferase)	Streptomycin, spectinomycin	Drug adenylylation = deactivation
***aadE***	TACCTTATTGCCCTTGGAAGAGTTA	GGAACTATGTCCCTTTTAATTCTACAATCT	ANT (adenylyltransferase)	Streptomycin, spectinomycin	Drug adenylylation = deactivation
***aphA1***	TGAACAAGTCTGGAAAGAAATGCA	CCTATTAATTTCCCCTCGTCAAAAA	APH (aminoglycoside phosphotransferase)	Aminoglycosides	Drug phosphorylation = deactivation
***str***	AATGAGTTTTGGAGTGTCTCAACGTA	AATCAAAACCCCTATTAAAGCCAAT	APH (aminoglycoside phosphotransferase)	Streptomycin	Drug phosphorylation = deactivate
***strA***	CCGGTGGCATTTGAGAAAAA	GTGGCTCAACCTGCGAAAAG	APH (aminoglycoside phosphotransferase)	Streptomycin	Drug phosphorylation = deactivate
***strB***	GCTCGGTCGTGAGAACAATCT	CAATTTCGGTCGCCTGGTAGT	APH (aminoglycoside phosphotransferase)	Streptomycin	Drug phosphorylation = deactivation
***ampC-01***	TGGCGTATCGGGTCAATGT	CTCCACGGGCCAGTTGAG	AmpC β-lactamase	Cephalosporins (esp. 2nd–3rd gen), penicillins	Enzymatic hydrolysis = deactivation
***ampC/blaDHA***	TGGCCGCAGCAGAAAGA	CCGTTTTATGCACCCAGGAA	AmpC β-lactamase (DHA family)	Cephalosporins (esp. 3rd gen), penicillins	β-lactam hydrolysis = deactivation
***bla1***	GCAAGTTGAAGCGAAAGAAAAGA	TACCAGTATCAATCGCATATACACCTAA	β-lactamase	Penicillins	β-lactam hydrolysis
***blaCMY2-01***	AAAGCCTCAT GGGTGCATAAA	ATAGCTTTTGTTTGCCAGCATCA	AmpC β-lactamase (CMY-2 family)	Cephalosporins (incl. 3rd gen), penicillins	β-lactam hydrolysis = deactivation
***blaCTX-M-04***	CTTGGCGTTGCGCTGAT	CGTTCATCGGCACGGTAGA	SBL (CTX-M family)	Extended-spectrum cephalosporins	β-lactam hydrolysis = deactivation
***blaCTX-M-05***	GCGATAACGTGGCGATGAAT	GTCGAGACGGAACGTTTCGT	SBL (CTX-M family)	Extended-spectrum cephalosporins	β-lactam hydrolysis = deactivation
***blaGES***	GCAATGTGCTCAACGTTCAAG	GTGCCTGAGTCAATTCTTTCAAAG	β-lactamase (GES family; some variants are ESBL, some carbapenemase)	Cephalosporins ± carbapenems	β-lactam hydrolysis = deactivation
***blaOXY***	CGTTCAGGCGGCAGGTT	GCCGCGATATAAGATTTGAGAATT	β-lactamase (OXY family)	Cephalosporin, penicillins	β-lactam hydrolysis = deactivation
***blaSFO***	CCGCCGCCATCCAGTA	GGGCCGCCAAGATGCT	ESBL (SFO-1 family)	Extended-spectrum cephalosporins	β-lactam hydrolysis = deactivation
***blaTEM***	AGCATCTTACGGATGGCATGA	TCCTCCGATCGTTGTCAGAAGT	β-lactamase (TEM family; many ESBL variants)	Penicillins ± cephalosporins	β-lactam hydrolysis = deactivation
***blaZ***	GGAGATAAAGTAACAAATCCAGTTAGATATGA	TGCTTAATTTTCCATTTGCGATAAG	β-lactamase (penicillinase)	Penicillins	β-lactam hydrolysis = deactivation
***cphA-01***	GCGAGCTGCACAAGCTGAT	CGGCCCAGTCGCTCTTC	MBL (metallo-β-lactamase, CphA family)	Carbapenems	β-lactam hydrolysis (Zn-dependent)
***mecA***	GGTTACGGACAAGGTGAAATACTGAT	TGTCTTTTAATAAGTGAGGTGCGTTAATA	PBP2a (altered penicillin-binding protein)	β-lactams (esp. methicillin, oxacillin)	Target alteration (low-affinity PBP)
***Pbp5***	GGCGAACTTCTAATTAATCCTATCCA	CGCCGATGACATTCTTCTTATCTT	Low-affinity PBP (penicillin-binding protein)	β-lactams	Target alteration (reduced PBP affinity)
***carB***	GGAGTGAGGCTGACCGTAGAAG	ATCGGCGAAACGCACAAA	Macrolide esterase	Macrolides (esp. erythromycin)	Drug hydrolysis (esterase)
***ereA***	CCTGTGGTACGGAGAATTCATGT	ACCGCATTCGCTTTGCTT	Macrolide esterase	Macrolides (esp. erythromycin)	Drug hydrolysis (esterase)
***ereB***	GCTTTATTTCAGGAGGCGGAAT	TTTTAAATGCCACAGCACAGAATC	Macrolide esterase	Macrolides	Drug hydrolysis (esterase)
***ermA***	TTGAGAAGGGATTTGCGAAAAG	ATATCCATCTCCACCATTAATAGTAAACC	rRNA methyltransferase (MLS_B)	Macrolides, lincosamides, streptogramin B	Target methylation (23S rRNA)
***ermA/ermTR***	ACATTTTACCAAGGAACTTGTGGAA	GTGGCATGACATAAACCTTCATCA	rRNA methyltransferase (MLS_B)	Macrolides, lincosamides, streptogramin B	Target methylation (23S rRNA)
***ermB***	TAAAGGGCATTTAACGACGAAACT	TTTATACCTCTGTTTGTTAGGGAATTGAA	rRNA methyltransferase (MLS_B)	Macrolides, lincosamides, streptogramin B	Target methylation (23S rRNA)
***ermC***	TTTGAAATCGGCTCAGGAAAA	ATGGTCTATTTCAATGGCAGTTACG	rRNA methyltransferase (MLS_B)	Macrolides, lincosamides, streptogramin B	Target methylation (23S rRNA)
***ermF***	CAGCTTTGGTTGAACATTTACGAA	AAATTCCTAAAATCACAACCGACAA	rRNA methyltransferase (MLS_B)	Macrolides, lincosamides, streptogramin B	Target methylation (23S rRNA)
***ermK-01***	GTTTGATATTGGCATTGTCAGAGAAA	ACCATTGCCGAGTCCACTTT	rRNA methyltransferase (MLS_B)	Macrolides, lincosamides, streptogramin B	Target methylation (23S rRNA)
***ermX***	GCTCAGTGGTCCCCATGGT	ATCCCCCCGTCAACGTTT	rRNA methyltransferase (MLS_B)	Macrolides, lincosamides, streptogramin B	Target methylation (23S rRNA)
***ermY***	TTGTCTTTGAAAGTGAAGCAACAGT	TAACGCTAGAGAACGATTTGTATTGAG	rRNA methyltransferase (MLS_B)	Macrolides, lincosamides, streptogramin B	Target methylation (23S rRNA)
***matA/mel***	TAGTAGGCAAGCTCGGTGTTGA	CCTGTGCTATTTTAAGCCTTGTTTCT	Efflux pump (ABC-type; MLS resistance)	Macrolides	Drug efflux
***mdtA***	CCTAACGGGCGTGACTTCA	TTCACCTGTTTCAAGGGTCAAA	Efflux pump (RND/ABC-associated, depending on operon)	Multidrug (various classes; species-dependent)	Drug efflux
***sul2***	TCATCTGCCAAACTCGTCGTTA	GTCAAAGAACGCCGCAATGT	Sulfonamide-resistant DHPS	Sulfonamides	Target alteration (drug-insensitive DHPS)
***sul1***	CAGCGCTATGCGCTCAAG	ATCCCGCTGCGCTGAGT	Sulfonamide-resistant DHPS	Sulfonamides	Target alteration (drug-insensitive DHPS)
***dfrA1***	GGAATGGCCCTGATATTCCA	AGTCTTGCGTCCAACCAACAG	Trimethoprim-resistant DHFR	Trimethoprim	Target alteration (drug-insensitive DHFR)
***folA***	CGAGCAGTTCCTGCCAAAG	CCCAGTCATCCGGTTCATAATC	DHFR (chromosomal dihydrofolate reductase)	Trimethoprim (when mutated)	Target alteration (reduced TMP binding)
***tet(32)***	CCATTACTTCGGACAACGGTAGA	CAATCTCTGTGAGGGCATTTAACA	Ribosomal protection protein (RPP)	Tetracyclines	Target protection (ribosome shielding)
***tet(34)***	CTTAGCGCAAACAGCAATCAGT	CGGTGATACAGCGCGTAAACT	Ribosomal protection protein (RPP)	Tetracyclines	Target protection (ribosome shielding)
***tet(35)***	ACCCCATGACGTACCTGTAGAGA	CAACCCACACTGGCTACCAGTT	Ribosomal protection protein (RPP)	Tetracyclines	Target protection (ribosome shielding)
***tetA-01***	GCTGTTTGTTCTGCCGGAAA	GGTTAAGTTCCTTGAACGCAAACT	Efflux pump (TetA family)	Tetracyclines	Drug efflux
***tetB-01***	AGTGCGCTTTGGATGCTGTA	AGCCCCAGTAGCTCCTGTGA	Efflux pump (TetB family)	Tetracyclines	Drug efflux
***tetC-01***	CATATCGCAATACATGCGAAAAA	AAAGCCGCGGTAAATAGCAA	Efflux pump (TetC family)	Tetracyclines	Drug efflux
***tetD-01***	TGCCGCGTTTGATTACACA	CACCAGTGATCCCGGAGATAA	Efflux pump (TetC family)	Tetracyclines	Drug efflux
***tetE***	TTGGCGCTGTATGCAATGAT	CGACGACCTATGCGATCTGA	Efflux pump (TetE family)	Tetracyclines	Drug efflux
***tetG-01***	TCAACCATTGCCGATTCGA	TGGCCCGGCAATCATG	Efflux pump (TetG family)	Tetracyclines	Drug efflux
***tetH***	TTTGGGTCATCTTACCAGCATTAA	TTGCGCATTATCATCGACAGA	Efflux pump (TetH family)	Tetracyclines	Drug efflux
***tetJ***	GGGTGCCGCATTAGATTACCT	TCGTCCAATGTAGAGCATCCATA	Efflux pump (TetJ family)	Tetracyclines	Drug efflux
***tetK***	CAGCAGTCATTGGAAAATTATCTGATTATA	CCTTGTACTAACCTACCAAAAATCAAAATA	Efflux pump (TetK family)	Tetracyclines	Drug efflux
***tetL-01***	AGCCCGATTTATTCAAGGAATTG	CAAATGCTTTCCCCCTGTTCT	Efflux pump (TetL family)	Tetracyclines	Drug efflux
***tetM-01***	CATCATAGACACGCCAGGACATAT	CGCCATCTTTTGCAGAAATCA	Efflux pump (TetM family)	Tetracyclines	Drug efflux
***tetO-01***	ATGTGGATACTACAACGCATGAGATT	TGCCTCCACATGATATTTTTCCT	Efflux pump (TetO family)	Tetracyclines	Drug efflux
***tetPA***	AGTTGCAGATGTGTATAGTCGTAAACTATCTATT	TGCTACAAGTACGAAAACAAAACTAGAA	Efflux pump (Tet(P)/TetPA family)	Tetracyclines	Drug efflux
***tetPB-01***	ACACCTGGACACGCTGATTTT	ACCGTCTAGAACGCGGAATG	Efflux pump (Tet(PB) family)	Tetracyclines	Drug efflux
***tetPB-02***	TGATACACCTGGACACGCTGAT	CGTCCAAAACGCGGAATG	Efflux pump (Tet(PB) family)	Tetracyclines	Drug efflux
***tetPB-03***	TGGGCGACAGTAGGCTTAGAA	TGACCCTACTGAAACATTAGAAATATACCT	Efflux pump (Tet(PB) family)	Tetracyclines	Drug efflux
***tetQ***	CGCCTCAGAAGTAAGTTCATACACTAAG	TCGTTCATGCGGATATTATCAGAAT	Ribosomal protection protein (RPP)	Tetracyclines	Target protection (ribosome shielding)
***tetR-02***	CGCGATAGACGCCTTCGA	TCCTGACAACGAGCCTCCTT	Transcriptional repressor (TetR family)	None directly	Regulation of tet efflux genes
***tetS***	TTAAGGACAAACTTTCTGACGACATC	TGTCTCCCATTGTTCTGGTTCA	Ribosomal protection protein (RPP)	Tetracyclines	Target protection (ribosome shielding)
***tetT***	CCATATAGAGGTTCCACCAAATCC	TGACCCTATTGGTAGTGGTTCTATTG	Efflux pump (TetT family)	Tetracyclines	Drug efflux
***tetU-01***	GTGGCAAAGCAACGGATTG	TGCGGGCTTGCAAAACTATC	Efflux pump (TetU family)	Tetracyclines	Drug efflux
***tetV***	GCGGGAACGACGATGTATATC	CCGCTATCTCACGACCATGAT	Efflux pump (TetV family)	Tetracyclines	Drug efflux
***tetW-01***	ATGAACATTCCCACCGTTATCTTT	ATATCGGCGGAGAGCTTATCC	Ribosomal protection protein (RPP)	Tetracyclines	Target protection (ribosome shielding)
***tetX***	AAATTTGTTACCGACACGGAAGTT	CATAGCTGAAAAAATCCAGGACAGTT	Flavin-dependent monooxygenase	Tetracyclines	Drug oxidation
***vanA***	AAAAGGCTCTGAAAACGCAGTTAT	CGGCCGTTATCTTGTAAAAACAT	Vancomycin resistance operon (D-Ala–D-Lac ligase system)	Glycopeptides (vancomycin, teicoplanin)	Target alteration (D-Ala → D-Lac cell wall precursor)
***vanB-01***	TTGTCGGCGAAGTGGATCA	AGCCTTTTTCCGGCTCGTT	Vancomycin resistance operon (D-Ala–D-Lac ligase system)	Vancomycin	Target alteration (D-Ala → D-Lac)
***vanC1***	AGGCGATAGCGGGTATTGAA	CAATCGTCAATTGCTCATTTCC	Vancomycin resistance operon (D-Ala–D-Ser ligase system)	Vancomycin	Target alteration (D-Ala → D-Ser)
***vanG***	ATTTGAATTGGCAGGTATACAGGTTA	TGATTTGTCTTTGTCCATACATAATGC	Vancomycin resistance operon (D-Ala–D-Ser ligase system)	Vancomycin	Target alteration (D-Ala → D-Ser)
***vanRA-01***	CCCTTACTCCCACCGAGTTTT	TTCGTCGCCCCATATCTCAT	Vancomycin resistance regulatory protein (VanR, response regulator)	None directly (regulates van operon)	Two-component regulation of van genes
***vanRB***	GCCCTGTCGGATGACGAA	TTACATAGTCGTCTGCCTCTGCAT	Vancomycin resistance regulatory protein (VanR, response regulator)	None directly (regulates van operon)	Regulation of van operon (two-component system)
***vanSA***	CGCGTCATGCTTTCAAAATTC	TCCGCAGAAAGCTCAATTTGTT	Sensor kinase (VanS)	None directly (regulates van operon)	Senses glycopeptides; regulates van operon (two-component system)
***vanSC-01***	ATCAACTGCGGGAGAAAAGTCT	TCCGCTGTTCCGCTTCTT	Sensor kinase (VanS family)	None directly (regulates van operon)	Regulation of van operon (glycopeptide sensing)
***vanTE***	GTGGTGCCAAGGAAGTTGCT	CGTAGCCACCGCAAAAAAAT	D-Ala–D-Ser ligase (VanT family component)	Glycopeptides (vancomycin)	Target alteration (D-Ala → D-Ser synthesis)
***vanXA***	CGCTAAATATGCCACTTGGGATA	TCAAAAGCGATTCAGCCAACT	D-Ala–D-Lac dipeptidase (VanX family)	None directly (part of van operon resistance)	Cleaves D-Ala–D-Ala to enforce D-Ala–D-Lac pathway (target alteration support)
***acrA-01***	CAACGATCGGACGGGTTTC	TGGCGATGCCACCGTACT	RND efflux pump component (AcrAB-TolC system)	Multidrug (β-lactams, quinolones, chloramphenicol, tetracyclines, etc.)	Drug efflux
***ceoA***	ATCAACACGGACCAGGACAAG	GGAAAGTCCGCTCACGATGA	RND efflux pump component (CeoAB-OmpN system)	Multidrug (quinolones, chloramphenicol, tetracyclines, etc.)	Drug efflux
***cmr***	CGGCATCGTCAGTGGAATT	CGGTTCCGAAAAAGATGGAA	Efflux pump (CMR/MFS-type)	Chloramphenicol	Drug efflux
***mexA***	AGGACAACGCTATGCAACGAA	CCGGAAAGGGCCGAAAT	RND efflux pump component (MexAB-OprM system)	Multidrug (β-lactams, quinolones, macrolides, tetracyclines, etc.)	Drug efflux
***mexF***	CCGCGAGAAGGCCAAGA	TTGAGTTCGGCGGTGATGA	RND efflux pump component (MexEF-OprN system)	Multidrug (quinolones, chloramphenicol, trimethoprim, etc.)	Drug efflux
***bla_OXA48_***	GCGTGGTTAAGGATGAACAC	CATCAAGTTCAACCCAACCG	Carbapenemase (OXA-48 family)	Carbapenems ± penicillins (weak ESBL activity)	β-lactam hydrolysis
***bla_KPC_***	ATGTCACTGTATCGCCGTCT	TTTTCAGAGCCTTACTGCCC	Carbapenemase (KPC family)	Carbapenems, cephalosporins, penicillins	β-lactam hydrolysis
***NDM***	GCAAATGGAAACTGGCGACC	TACCGCCCATCTTGTCCTGA	Carbapenemase (metallo-β-lactamase; NDM family)	Carbapenems, cephalosporins, penicillins	β-lactam hydrolysis (Zn-dependent)
***qnrB***	AGGACAACGCTATGCAACGAA	GGATATCTAAATCGCCCAGTTCC	Qnr (PMQR protein)	Fluoroquinolones	Target protection (gyrase/topo IV shielding)
***MDFa***	GCATTGATTGGGTTCCTAC	CGCGGTGATCTTGATACA	MFS efflux pump	Multidrug (tetracyclines, chloramphenicol, fluoroquinolones, macrolides, etc.)	Drug efflux
***emrE***	TATTTATCTTGGTGGTGCAATAC	ACAATACCGACTCCTGACCAG	SMR efflux pump (small multidrug resistance)	Multidrug (quaternary ammonium, dyes; low-level to some antibiotics)	Drug efflux
***qacE*** ***Δ*** ***1***	AATCCATCCCTGTCGGTGTT	CGCAGCGACTTCCACGATGGGGAT	SMR efflux pump (quaternary ammonium compound resistance)	None directly (biocides/disinfectants)	Efflux of QACs
***qacC***	GGCTTTTCAAAATTTATACCATCCT	ATGCGATGTTCCGAAAATGT	SMR efflux pump	None directly (QACs/disinfectants)	Drug efflux
***VanA***	GCCGGAAAAAGGCTCTGAA	TTTTTTGCCGTTTCCTGTATCC	Vancomycin resistance operon (D-Ala–D-Lac ligase system)	Glycopeptides (vancomycin, teicoplanin)	Target alteration (D-Ala → D-Lac)
***VanB***	GATTTGATTGTCGGCGAAGTG	TCCTGATGGATGCGGAAGA	Vancomycin resistance operon (D-Ala–D-Lac ligase system)	Vancomycin (variable teicoplanin)	Target alteration (D-Ala → D-Lac)
***blaSHV***	GGTTATGCGTTATATTCGCC	TTAGGTTGCCAGTGCTC	Class A β-lactamase (ESBL in some variants)	Class A β-lactamase (ESBL in some variants)	Class A β-lactamase (ESBL in some variants)
***blaPER-1***	AATTTGGGCTTAGGGCAGAA	ATGAATGTCATTATAAAAGC	ESBL (PER family)	ESBL (PER family)	ESBL (PER family)
***blaOXA-1***	TTTTCTGTTGTTTGGGTTTT	TTTCTTGGCTTTTATGCTTG	Narrow-spectrum OXA β-lactamase	Narrow-spectrum OXA β-lactamase	Narrow-spectrum OXA β-lactamase
***blaOXA-10***	ATTATCGGCCTAGAAACTGG	CTTACTTCGCCAACTTCTCTG	OXA family β-lactamase	OXA family β-lactamase	OXA family β-lactamase
***blaIMP***	GTACGCATCACCGTCGACAC	TGACGGGACGTATACAACCAGA	Metallo-β-lactamase (IMP family)	Metallo-β-lactamase (IMP family)	Metallo-β-lactamase (IMP family)
***blaVIM***	GGTGCTGCGCATTCGACCGACA	TTCGGTCCAGTAGAACTCTTCTATCC-	Metallo-β-lactamase (VIM family)	Metallo-β-lactamase (VIM family)	Metallo-β-lactamase (VIM family)
***blaFOX-1***	ATGCAACAACGACGTGCG	TCACTCGGCCAACTGACT	AmpC β-lactamase (FOX family)	AmpC β-lactamase (FOX family)	AmpC β-lactamase (FOX family)
***aac(3)-IIa***	CGGAAGGCAATAACGGAG	TCGAACAGGTAGCACTGAG	Aminoglycoside acetyltransferase	Aminoglycoside acetyltransferase	Aminoglycoside acetyltransferase
***aac(3)-IV***	GTGTGCTGCTGGTCCACAGC	AGTTGACCCAGGGCTGTCGC	Aminoglycoside acetyltransferase	Aminoglycoside acetyltransferase	Aminoglycoside acetyltransferase
***aac(6′)-Ib-cr***	TTGCGATGCTCTATGAGTGGCTA	CTCGAATGCCTGGCGTGTTT	Bifunctional AAC (also affects quinolones)	Bifunctional AAC (also affects quinolones)	Bifunctional AAC (also affects quinolones)
***aph(3′)-IIIa***	AAATACCGCTGCGTA	CATACTCTTCCGAGCAA	Aminoglycoside phosphotransferase	Aminoglycoside phosphotransferase	Aminoglycoside phosphotransferase
***aadA2***	TGCGACTCGATCCTCGATCTG	ACCAGCTCGAATGCCTGGC	Aminoglycoside nucleotidyltransferase	Aminoglycoside nucleotidyltransferase	Aminoglycoside nucleotidyltransferase
***qnrS***	TGCAGAAAGCCAGAAATTGC	GATCGTTGGAAGTATTGAAC	Qnr family (PMQR)	Qnr family (PMQR)	Qnr family (PMQR)
***qnrVC***	GCTCAAACCTCCGAGATACAC	AAGCATCTCGAAGATCAGCAT	Qnr family (PMQR)	Qnr family (PMQR)	Qnr family (PMQR)
***oqxA***	CTCGGCGCGATGATGCT	CCACTCTTCACGGGAGACGA	RND efflux component (OqxAB)	RND efflux component (OqxAB)	RND efflux component (OqxAB)
***qepA***	GCAGGTCCAGCAGCGGGTAG	CTTCCTGCCCGAGTATCGTG	MFS efflux pump	MFS efflux pump	MFS efflux pump
***ermT***	TCAAATCGAGGTAGCAAAATAG	CCACCTGTT AGCCTTATTACT	rRNA methyltransferase (MLS_B)	RNA methyltransferase (MLS_B)	RNA methyltransferase (MLS_B)
***mefA***	TTGCGATGCTCTATGAGTGGCTA	CTCGAATGCCTGGCGTGTTT	Macrolide efflux (MFS)	Macrolide efflux (MFS)	Macrolide efflux (MFS)
***msrA***	GCCCAAAAGTGGGATAACAGA	GCAACACGAAATGCAGAACC	ABC-F protection protein	ABC-F protection protein	ABC-F protection protein
***lnuA***	GGTGGCTGGGGGGTAGATGTATTAACTGG	GCTTCTTTTGAAATACATGGTATTTTTCGATC	Lincosamide nucleotidyltransferase	Lincosamide nucleotidyltransferase	Lincosamide nucleotidyltransferase
***lnuB***	ATGAAAGGGTGAAGAAATGTTA	CGTTACTCTCCTATTCACTAATGT	Lincosamide nucleotidyltransferase	Lincosamide nucleotidyltransferase	Lincosamide nucleotidyltransferase
***tetA(A)***	CCGCGCTTTGGGTCATT	TGGTCGCGTCCCAGTGA	Efflux pump (TetA family)	Efflux pump (TetA family)	Efflux pump (TetA family)
***tetB(P)***	GGAAAATACCGTTTTATGCA	ACTCCACTCCATTCTGAAAGC	Efflux pump	Efflux pump	Efflux pump
***tetY***	CAGCATCCAAAGCGCACTT	CTTATTGCTGGCTTTTT	Efflux pump	Efflux pump	Efflux pump
***tetZ***	CCTTCTCGACCAGGTCGG	ACCCACAGCGTGTCCGTC	Efflux pump	Efflux pump	Efflux pump
***tetW/N/W***	GAGAGCCTGCTATATGCCAGC	GGGCGTATCCACAATGTTAAC	Ribosomal protection protein	Ribosomal protection protein	Ribosomal protection protein
***tetX3***	CAGTGAAAATTCACTGGCAAC	ATCCAAAGTGTGGTTGAGAAT	Flavin-dependent monooxygenase	Flavin-dependent monooxygenase	Flavin-dependent monooxygenase
***tetX4***	ACGGARAGTTTATTGTATACC	TGGCGTATCTATAATGTTGAC	Flavin-dependent monooxygenase	Flavin-dependent monooxygenase	Flavin-dependent monooxygenase
***tet(36)***	AGAATCTGCTGTTTGCCAGTG	CGGAGTGTCAATGATATTGCA	Ribosomal protection protein	Ribosomal protection protein	Ribosomal protection protein
***sul3***	CATTCTAGAAAACAGTCGTAGTTCG	CATCTGCAGCTAACCTAGGGCTTTGGA	Sulfonamide-resistant DHPS	Sulfonamides	Target alteration (drug-insensitive DHPS)
***dfrA12***	TCAAAGATAAAC GGAATAGAC	ACCAGCTCG AATGCCTGGC	Trimethoprim-resistant DHFR	Trimethoprim	Target alteration
***dfrA17***	TCAAAGATAAACGGAATAGAC	ACCAGCTCG AATGCCTGGC	Trimethoprim-resistant DHFR	Trimethoprim	Target alteration
***dfrG***	TGCTGCGATGGATAAGAA	TTCTTATCCATCGCAGCA	Trimethoprim-resistant DHFR	Trimethoprim	Target alteration
***dfrK***	TGCTGCGATGGATAAGAAA	TTCTTATCCATCGCAGCAA	Trimethoprim-resistant DHFR	Trimethoprim	Target alteration
***mcr-1***	TTGCGATGCTCTATGAGTGGCTA	CTCGAATGCCTGGCGTGTTT	Phosphoethanolamine transferase	Colistin (polymyxin E)	Lipid A modification (reduced binding)
***mcr-2***	CGACCAAGCCGAGTCTAAGG	CAACTGCGACCAACACACTT	Phosphoethanolamine transferase	Colistin	Lipid A modification
***mcr-3***	ACCTCCAGCGTGAGATTGTTCCA	GCGGTTTCACCAACGACCAGAA	Phosphoethanolamine transferase	Colistin	Lipid A modification
***mcr-4***	AGAATGCCAGTCGTAACCCG	GCGAGGATCATAGTCTGCCC	Phosphoethanolamine transferase	Colistin	Lipid A modification
***mcr-5***	CTGTGGCCAGTCATGGATGT	CGAATGCCCGAGATGACGTA	Phosphoethanolamine transferase	Colistin	Lipid A modification
***mcr-8***	TCCGGGATGCGTGACGTTGC	TGCTGCGCGAATGAAGACGA	Phosphoethanolamine transferase	Colistin	Lipid A modification
***mcr-9***	CGGTACCGCTACCGCAATAT	ATAACAGCGAGACACCGGTT	Phosphoethanolamine transferase	Colistin	Lipid A modification
***mcr-10***	CCCACGCTGATGTTCCTG	AAGGCATTKGTRTCACGCG	Phosphoethanolamine transferase	Colistin	Lipid A modification
***acrB***	CGTACACAGAAAGTGCTCAA	CGCTTCAACTTTGTTTTCTT	RND efflux component (AcrAB-TolC)	Multidrug (β-lactams, quinolones, tetracyclines, chloramphenicol, dyes)	Drug efflux
***tolC***	AAGCCGAAAAACGCAACCT	CAGAGTCGGTAAGTGACCATC	Outer membrane channel (AcrAB-TolC)	Multidrug (partner of several RND/MFS systems)	Drug extrusion channel
***mexB***	GTGTTCGGCTCGCAGTACTC	AACCGTCGGGATTGACCTTG	RND efflux component (MexAB-OprM)	Multidrug	Drug efflux
***mexY***	CCGCTACAACGGCTATCCCT	AGCGGGATCGACCAGCTTTC	RND efflux component (AmrAB-OprA/MexXY)	Aminoglycosides, fluoroquinolones, etc.	Drug efflux
***smeD***	CGTCAAGGGAAAGGTATTAGCG	GTCGCCTTCATATTTTGCACCA	RND efflux component (SmeDEF)	Multidrug (S. maltophilia)	Drug efflux
***smeF***	GCCACGCTGAAGACCTA	CACCTTGTACAGGGTGA	RND efflux component (SmeDEF)	Multidrug	Drug efflux
***adeA***	CGCAAGTCGGAGGTATCATT	TATACCTGAGGCTCGCCACT	RND efflux component (AdeABC)	Multidrug (Acinetobacter)	Drug efflux
***adeB***	GGATTATGGCGACAGAAGGA	AATACTGCCGCCAATACCAG	RND efflux component (AdeABC)	Multidrug	Drug efflux
***adeJ***	CTCGGCGCGATGATGCT	CCACTCTTCACGGGAGACGA	RND efflux component (AdeIJK)	Multidrug	Drug efflux
***norA***	TTGCGATGCTCTATGAGTGGCTA	CTCGAATGCCTGGCGTGTTT	MFS efflux pump (Staphylococcus)	Fluoroquinolones, dyes	Drug efflux
***lmrP***	ATTGCGTAGCCATGTGCGTC	CTGTTTCTCCACCAATCAGCG	MFS efflux pump (Lactococcus/Enterococcus-like)	Macrolides, lincosamides, dyes	Drug efflux
***smeT***	GCAGCCTCGTTCACGCCTC	ATGGCCCGCAAGACCAAAGAG	TetR-family regulator (SmeDEF)	Indirectly mediates multidrug efflux	Transcriptional regulation
***vanD***	GCCATACTGGGAAAYGRAAA	CAGCCAAGTAYCCGGTAAATC	VanD-type ligase operon	Vancomycin (variable teicoplanin)	D-Ala–D-Lac ligase; target alteration
***vanE***	TGGATTCCTGCATCAACAGA	TTGCCAATGATAAACGCTGA	VanE-type ligase operon	Vancomycin	D-Ala–D-Ser ligase; target alteration
***vanL***	AAGTCAATAGCGCGGACGAA	GCGGCACTGTTTCCCAATAC	VanL-type ligase operon	Vancomycin	D-Ala–D-Ser ligase; target alteration
***vanN***	CCTCTGCCATTTGTATGAAT	TTGACGCTTGATTTCTCTAC	VanN-type ligase operon	Vancomycin	D-Ala–D-Ser ligase; target alteration
***qacA***	GCAGAAAGTGCAGAGTTCG	CCAGTCCAATCATGCCTG	MFS efflux pump (QAC resistance)	Quaternary ammonium compounds, some dyes	Efflux of QACs
***qacB***	GCAGAAAGTGCAGAGTTCG	CCAGTCCAATCATGCCTG	MFS efflux pump (QAC resistance)	Quaternary ammonium compounds	Efflux of QACs
***smr (qacC/qacD family)***	TGCAACACCTACCACTAAATATAACTT	CGAAACTACGCCGACTATGA	SMR efflux pump	QACs, intercalating dyes	Efflux of QACs
***qacH***	GCAACACCTACCACTAAATATAACTT	GCAACACCTACCACTAAATATAACTT	SMR efflux pump	QACs	Efflux of QACs
***qacJ***	TTGGGCAGGGTTAGGACTAG	TTGGGCAGGGTTAGGACTAG	SMR efflux pump	QACs	Efflux of QACs
***sugE(p)***	TTACCGTTACCGCCATGATT	TGAAACAGCAGATGCTGGAC	SMR-like efflux pump	QACs, dyes	Efflux
***bcrABC***	GAATGGATCCTTCAATTAGATCGAGGCACG	GTATGAATTCGTATAATCCGGATGCTGCCC	Bile salt/disinfectant resistance locus	Bile salts, some disinfectants (Listeria)	Efflux/membrane modification
***cepA*** **(e.g., *Enterococcus*)**	GCGGGCGGATATGCTTCATT	ATGCCAGCCGTACCAGGATA	Efflux pump	Chlorhexidine, QACs (species-dependent)	Efflux
***kpnEF***	GCCCTGTCATATATTTTTCTTGCCTTTGCG	GCCGCCAGGGACAGGATGCCGTAGATCTTG	Efflux pump (Klebsiella)	QACs, some antibiotics	Efflux
***adeRS***	AAAACGTGAAGGCATGAGTG	CTTCCCAACCGTTTAATTCG	Two-component regulator (AdeABC)	Indirectly to chlorhexidine and QAC tolerance via efflux	Regulation of efflux
***intI2***	GCGTTTTATGTCTAACAGTCC	AAGTAGCATCAGTCCATCC	Class 2 integron integrase	None directly (mobilizes gene cassettes)	Gene-cassette integration
***intI3***	TGGAGGTGCCTCCGGCAGCGAC	TTGCCAAACACGTATCTGTC	Class 3 integron integrase	None directly	Gene-cassette integration
***tnpA IS26***	TCGATAGATTGTCGCACCTG	CTGCCTCGGTGAGTTTTCTC	Transposase (IS26)	None directly (mobilizes resistance clusters)	Transposition/mobilization
***tnpM***	TCGTACATCGGCGGTGAAG	CCGCGAACCATGTGGTAAC	Transposition regulator	None directly	Regulates transposon activity
***orf513***	CTCACGCCCTGGCAAGGTTT	CTTTTGCCCTAGCTGCGG	Integron-associated recombinase-like	None directly	Gene-cassette recombination

**Table 3:** Detection summary across 10 ARG classes.

ARG Class	Number of Targets in Panel	Detected > 1	Median Genes per Sample (SR)	Median Genes per Sample (SBO)
**1. β-lactamases**	29	21	6	2
**2. Carbapenemases**	12	6	2	0
**3. Aminoglycoside**	18	9	3	1
**4. Quinolone (qnr, qep)**	16	10	3	1
**5. Macrolide/Lincosamide**	14	8	3	1
**6. Tetracycline (tet genes)**	22	19	5	2
**7. Sulfonamide/Trimethoprim**	13	11	3	1
**8. Glycopeptide (van genes)**	7	2	0–1	0
**9. Efflux pumps (mdf, emr, mex, nor)**	28	26	10	4
**10. Disinfectant (qac, smr)**	19	18	8	2

**Table 4:** The most commonly detected ARGs and the percentage of tourniquets contaminated by them.

Gene	Frequency (%)
***qacC***	47%
***mdf***	43%
***emr***	39%
***mexA***	26%
***mexF***	21%
***qnrB***	19%
***hace***	11%
***vanA***	6%
***oxa48***	4%
***blaKPC***	2%

**Table 5:** Comparison of the ARGs frequency by unit (SR—Emergency Department, SBO—Operating theater).

Gene	SR (*n* = 30)	SBO (*n* = 23)	*p*-Value
***qacC***	70%	17%	**<0.001**
***mdf***	63%	13%	**<0.001**
***emr***	57%	13%	**<0.01**
***mexA***	33%	17%	0.19
***mexF***	30%	9%	0.06
***qnrB***	27%	9%	0.12
***hace***	17%	4%	0.11
***vanA***	10%	0%	0.07
***oxa48***	7%	0%	0.18
***blaKPC***	3%	0%	0.33

Bold indicates statistically significant differences (*p* < 0.05).

## Data Availability

The present study did not generate novel nucleotide sequence data. Antibiotic and disinfectant resistance determinants were detected by targeted quantitative PCR using validated primer sets, without the sequencing of PCR amplicons. Therefore, no nucleotide sequence data deposition was required.

## References

[1] World Health Organization (2024). Global Report on Infection Prevention and Control 2024.

[2] Dall C. (2023). ECDC Estimates 4.3 Million Patients Get Healthcare-Associated Infections in European Hospitals. CIDRAP. https://www.cidrap.umn.edu/antimicrobial-stewardship/ecdc-estimates-43-million-patients-get-healthcare-associated-infections.

[3] Kleja M. (2024). Patient Safety Risk in EU: Healthcare-Associated Infections and Antibiotic Use Rise. Euractiv. https://www.euractiv.com/section/health-consumers/news/patient-safety-risk-in-eu-healthcare-associated-infections-and-antibiotic-use-rise/.

[4] McDonnell A., Countryman A., Laurence T., Gulliver S., Drake T., Edwards S., Kenny C., Lamberti O., Morton A., Shafira A. (2024). Forecasting the Fallout from AMR: Economic Impacts of Antimicrobial Resistance in Humans.

[5] Laurence T., Lamberti O., Smith R., Drake T., McDonnell A. (2025). The Global Direct Inpatient Cost of Antimicrobial Resistance: A Modelling Study.

[6] Rex J.H. (2024). Without Action, AMR Costs Go from $66 Billion to $159 Billion per Year by 2050. AMR Solutions. https://amr.solutions/2024/09/27/without-action-amr-costs-go-from-66b-to-159b-yr-by-2050/.

[7] Mauldin P.D., Salgado C.D., Hansen I.S., Durup D.T., Bosso J.A. (2010). Attributable hospital cost and length of stay associated with healthcare-associated infections caused by antibiotic-resistant Gram-negative bacteria. Antimicrob. Agents Chemother..

[8] Otieku E., Kurtzhals J.A.L., Fenny A.P., Ofori A.O., Labi A.K., Enemark U. (2024). Healthcare provider cost of antimicrobial resistance in two teaching hospitals in Ghana. Health Policy Plan..

[9] Szymczyk J., Kurpas M., Krasiński B., Zorena K., Mędrzycka-Dąbrowska W. (2025). Reusable tourniquets as potential transmitters of infection: A microbiological analysis. Microorganisms.

[10] Koczura R. Autoreferat Habilitacyjny (University of Lodz, 2020). https://www.biol.uni.lodz.pl/fileadmin/Wydzialy/Wydzia%C5%82_Biologii_i_Ochrony_%C5%9Arodowiska/Nauka_i_Badania/Habilitacje/w_latach_2016_-_2020/Ryszard_Koczura_habilitacja_autoreferat_15.pdf.

[11] Weber D.J., Rutala W.A., Anderson D.J., Sickbert-Bennett E.E. (2023). Biofilms on medical instruments and surfaces: Do they interfere with instrument reprocessing and surface disinfection?. Am. J. Infect. Control.

[12] Mishra A., Aggarwal A., Khan F. (2024). Medical device-associated infections caused by biofilm-forming microbial pathogens and controlling strategies. Antibiotics.

[13] Liu Y., Huang S., Zhou J., Zhang C., Hu F., Xiao Y., Qiu H., Yang Y. (2022). A new method for the rapid detection of the antibacterial and bacteriostatic activity of disinfectants based on Propidium Monoazide combined with real-time PCR. Front. Microbiol..

[14] Global AMR R&D Hub (2025). One Health. https://globalamrhub.org/one-health/.

[15] Field N., Cohen T., Struelens M.J., Palm D., Cookson B., Glynn J.R., Gallo V., Ramsay M., Sonnenberg P., MacCannell D. (2014). Strengthening the reporting of molecular epidemiology for infectious diseases (STROME-ID). Lancet Infect. Dis..

[16] Szymczyk J., Jaskulak M., Kurpas M., Zorena K., Mędrzycka-Dąbrowska W. (2025). Metagenomic analysis of bacterial diversity on reusable tourniquets in hospital environments. Appl. Sci..

[17] Jenedi K., Temiz M., Duran N., Duran G.G., Eryılmaz N. (2014). Relationship between resistance genes to quaternary ammonium compounds and antibiotic resistance in staphylococci from surgical site infections. Med. Sci. Monit..

[18] Buffet-Bataillon S., Tattevin P., Bonnaure-Mallet M., Jolivet-Gougeon A. (2012). Emergence of resistance to antibacterial agents: The role of quaternary ammonium compounds—a critical review. Int. J. Antimicrob. Agents.

[19] Hanafiah A., Sukri A., Yusoff H., Chan C.S., Hazrin-Chong N.H., Salleh S.A., Neoh H.-M. (2024). Insights into the Microbiome and Antibiotic Resistance Genes from Hospital Environmental Surfaces: A Prime Source of Antimicrobial Resistance. Antibiotics.

[20] Boyce J.M., Havill N.L. (2022). In-use contamination of a hospital-grade disinfectant. Am. J. Infect. Control.

[21] Zendri F., Isgren C.M., Devaney J., Schmidt V., Rankin R., Timofte D. (2023). Resistome-based surveillance identifies ESKAPE pathogens as the predominant gram-negative organisms circulating in veterinary hospitals. Front. Microbiol..

[22] Ma X., Dong X., Cai J., Fu C., Yang J., Liu Y., Zhang Y., Wan T., Lin S., Lou Y. (2022). Metagenomic Analysis Reveals Changes in Bacterial Communities and Antibiotic Resistance Genes in an Eye Specialty Hospital and a General Hospital Before and After Wastewater Treatment. Front. Microbiol..

[23] Grohmann M., Schomakers L., Wolschendorf F., Grosch J., Lindner S., Witte A.K. (2020). Reduced bacterial contamination rates detected on silicone tourniquets compared to conventional tourniquets in clinical routine. BMC Infect. Dis..

[24] Duller S., Kumpitsch C., Moissl-Eichinger C., Wink L., Mora K.K., Mahnert A. (2024). In-hospital areas with distinct maintenance and staff/patient traffic have specific microbiome profiles, functions, and resistomes. mSystems.

